# Tensile Yield Strain of Human Cortical Bone from the Femoral Diaphysis Is Constant among Healthy Adults and across the Anatomical Quadrants

**DOI:** 10.3390/bioengineering11040395

**Published:** 2024-04-19

**Authors:** Massimiliano Baleani, Paolo Erani, Alice Acciaioli, Enrico Schileo

**Affiliations:** 1 Laboratorio di Tecnologia Medica, IRCCS Istituto Ortopedico Rizzoli, 40136 Bologna, Italy; paolo.erani@ior.it (P.E.); alice.acciaioli@ior.it (A.A.); 2 Laboratorio di Bioingegneria Computazionale, IRCCS Istituto Ortopedico Rizzoli, 40136 Bologna, Italy; enrico.schileo@ior.it

**Keywords:** human cortical bone, femur, tensile tests, yield strain, yield stress, elastic modulus

## Abstract

The literature suggests that the yield strain of cortical bone is invariant to its stiffness (elastic modulus) and strength (yield stress). However, data about intra-individual variations, e.g., the influence of different collagen/mineral organisations observed in bone aspects withstanding different habitual loads, are lacking. The hypothesis that the yield strain of human cortical bone tissue, retrieved from femoral diaphyseal quadrants subjected to different habitual loads, is invariant was tested. Four flat dumbbell-shaped specimens were machined from each quadrant of the proximal femoral diaphysis of five adult donors for a total of 80 specimens. Two extensometers attached to the narrow specimen region were used to measure deformation during monotonic tensile testing. The elastic modulus (linear part of the stress–strain curve) and yield strain/stress at a 0.2% offset were obtained. Elastic modulus and yield stress values were, respectively, in the range of 12.2–20.5 GPa and 75.9–136.6 MPa and exhibited a positive linear correlation. All yield strain values were in the narrow range of 0.77–0.87%, regardless of the stiffness and strength of the tissue and the anatomical quadrant. In summary, the results corroborate the hypothesis that tensile yield strain in cortical bone is invariant, irrespective also of the anatomical quadrant. The mean yield strain value found in this study is similar to what was reported by inter-species and evolution studies but slightly higher than previous reports in humans, possibly because of the younger age of our subjects. Further investigations are needed to elucidate a possible dependence of yield strain on age.

## 1. Introduction

The strength of bone segments is determined by the size, shape, and properties of the constituent material. In the femur, which is the biggest and clinically most relevant human long bone, cortical bone has been reported to be the major contributor to whole bone strength [1,2]. A full understanding of the material properties of bone tissue and the bases of bone strength/fragility is still a major research challenge, requiring investigations over the whole hierarchical tissue structure and its interaction in fracture mechanisms [3].

The size and shape of the femur, although quite variable among individuals [4,5], can be effectively captured through imaging diagnostics to, e.g., initialise personalised numerical models aimed to estimate bone strength [6,7,8]. Cortical bone material properties in the elastic regime, known to be anisotropic and inhomogeneous, can also be incorporated in image-based personalised numerical models as follows: (i) the elastic modulus along the major axis is strongly dependent on density ([9], see also [10] for a review of earlier works), whose local variation can be modelled from quantitative imaging diagnostics, and (ii) anisotropy ratios, as determined by state-of-the-art ultrasound tests, can be approximated by relatively simple micromechanical models [11]. The incorporation of post-elastic and failure properties in numerical models remains more of a concern, with great variability in approach and degree of complexity among published models [12].

Each fracture criterion stems from the definition of an elastic limit (also known as yield point) envelope to then form a post-yield/failure law. Additionally, knowledge of cortical bone yield properties has a general interest per se, as it is key to understanding how far a bone is to exhaust its physiological strain reservoir under loading. According to the literature, looking at strain rather than stress measures to define the elastic limit of bone tissue brings the advantage of a weaker dependence from apparent density. This has received several confirmations for trabecular bone [3] and may also hold valid in cortical bone, where yield strain has shown the least inter-subject and inter-species variability among several mechanical properties [13]. When experimentally measuring yield strain, tensile testing would be preferable: with respect to compressive testing, because it directly challenges cortical bone toughening mechanisms [14] mostly related to the collagen phase (in fact bone tensile yield values are generally lower than compressive ones [3]); with respect to the common three-point bending testing because, in a heterogeneous material such as cortical bone, tensile testing maintains a homogeneous deformation within the specimen measurement length.

The literature data on the determination of the tensile yield strain of human cortical bone from tensile tests suggest a rather robust invariance in yield strain around the value of 0.07%. However, to our knowledge, this value comes from a few reports including two extensive studies [15,16] plus one single specimen within a larger inter-species investigation [17]. A fourth study performed tensile testing of human femoral cortical bone specimens but concentrated on the dependence of yield strain on the applied strain rate [18]. One aspect not addressed in those reports is intra-individual variability, as Mirzaali et al. [16] tested a single specimen per donor in tension, and all specimens were taken from the antero-lateral quadrant, and Bayraktar et al. [15] did not report per donor sample size or details of the anatomical aspects from which specimens were excised. Hansen et al. [18] retrieved 50 dumbbell-shaped specimens, also known as dog-bone-shaped specimens, from the two femurs of the same donor. Therefore, the anatomical aspect was neglected. A possible dependence of yield strain on the anatomical quadrant deserves a dedicated study. In fact, it has been hypothesised that the variable orientation of collagen fibres within osteons among bone anatomical quadrants is associated with different habitual stress states bone regions undergo [19], and this may be consistent with variations in the mechanical anisotropy ratios among femoral quadrants [20]. Thus, our aim in this study was to test the hypothesis that in the proximal femoral diaphysis (a bone portion stressed inhomogeneously around its periphery and featuring a cortical wall thick enough to extract tensile bone specimens), the yield strain values measured during tensile tests are indeed invariant among individuals and anatomical quadrants.

## 2. Materials and Methods

### 2.1. Slicing the Proximal Diaphysis of the Femur

Five fresh frozen femurs were obtained from an international donor program. The femurs were obtained from five male donors (mean age: 50 years, range: 34–66 years) with no history of musculoskeletal disorders. Each femur was cleaned of all soft tissues. A reference system was marked on the femur to identify the frontal plane, the sagittal plane, and the longitudinal axis of the femur [21]. This reference system ensured a repeatable spatial orientation of the femur.

**Figure 1:**
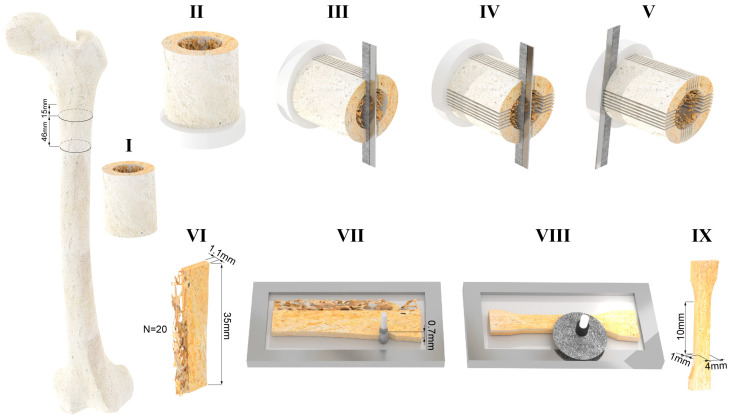
(**I**) A 46 mm thick slice is cut from the proximal femoral diaphysis. (**II**) The distal end of the diaphyseal slice is embedded in acrylic resin. (**III**) Six cuts are made in the proximal–distal direction, parallel to the frontal plane of the femur. (**IV**) Six cuts are made in the proximal–distal direction, parallel to the sagittal plane of the femur. (**V**) A cut is made orthogonal to the axis of the slice at 35 mm from the proximal end. (**VI**) Sketch showing the dimensions of the cortical slices that were obtained. (**VII**) The contour of the narrow part of the sample is machined with a diamond-coated tool. The periosteal-side contour of the narrow section was machined 0.7 mm inside the original periosteal surface. (**VIII**) The surface of the flat dumbbell-shaped specimen is polished by removing 0.05 mm of cortical bone tissue. (**IX**) Sketch showing the dimensions of the narrow part of flat dumbbell-shaped specimens that were obtained (note: because of the local anatomy of the cortical wall, non-standard (i.e., smaller with a 10:1 ratio of gauge length to thickness and 2.5:1 ratio of gauge length to width) dumbbell specimens were obtained).

The femur was clamped distally in a vice mounted on a dovetail slide controlled by a micrometre screw, and a 46 mm thick diaphyseal slice was cut from the femoral diaphysis 15 mm below the lesser trochanter (Figure 1I). Cutting was performed under constant water irrigation with a cut-off machine (Remet TR60, Remet, Bologna, Italy) equipped with a diamond abrasive cutting wheel. A sub-trochanteric resection level was chosen for two main reasons as follows: (i) the cortical bone tissue of each quadrant is subjected to different habitual loading conditions, i.e., the value of the experienced principal strains varies from quadrant to quadrant [22,23,24,25], and (ii) the diaphyseal cortical wall below the lesser trochanter is expected to be thicker than 4 mm [26].

**Figure 2:**
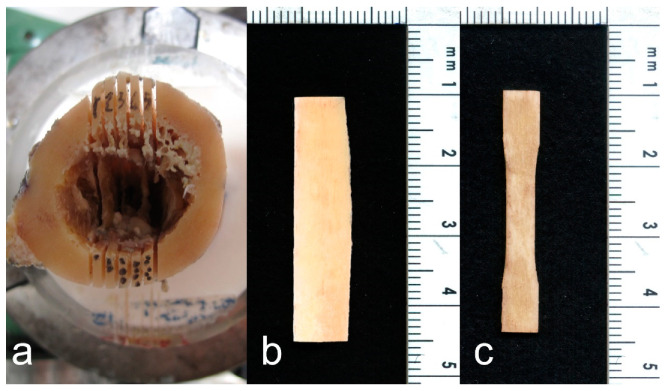
(**a**) The diaphyseal slice with six cuts in the proximal–distal direction, parallel to the frontal plane of the femur (step III in Figure 1). (**b**) A cortical slice retrieved from the cortical wall (step VI in Figure 1). No trabecular bone tissue is present on the endosteal side. (**c**) The flat dumbbell-shaped specimen obtained at the end of the procedure (step IX in Figure 1). The periosteal side (left edge of the specimen) of the gripping part was not machined. Therefore, irregularities in the left contour of the gripping part of the specimen are visible.

The distal end of the diaphyseal slice was embedded in acrylic resin poured into a cylindrical container, to a depth of 10 mm (Figure 1II). The resin cylinder was used to constrain the distal end of the diaphyseal slice in a dividing head; the longitudinal axis of the diaphyseal slice was parallel to the axis of the dividing head. The dividing head was mounted on a dovetail slide with a micrometre screw of a band saw (Exakt-311, EXAKT Advanced Technologies GmbH, Norderstedt, Germany) equipped with a diamond-coated band saw blade. The axis of the dividing head was orthogonal to the saw blade. The dovetail slide allowed for the dividing head position to be adjusted horizontally, i.e., the slice thickness could be set to the desired value. The angular position of the dividing head was adjusted to align the frontal plane marked on the diaphyseal slice vertically, i.e., parallel to the saw band. Six parallel cuts were made symmetrically to the frontal plane in increments of 1.1 mm plus the band saw thickness (0.8 mm) (Figure 1III, Figure 2a). Each cut was 36 mm deep, i.e., the saw band slightly touched the surface of the resin cylinder at the end of each cut. After completing the six-cut series, the angular position of the dividing head was rotated by 90 degrees to align the sagittal plane vertically, i.e., parallel to the saw band. Six further cuts were made as described above (Figure 1IV). The original position of each of the 20 cortical (1.1 mm thick) slices was marked on the proximal end of each slice. The dividing head was rotated 90 degrees around a vertical axis. A cut was then made orthogonal to the diaphyseal slice axis 35 mm apart from the proximal end (Figure 1V). Four specimens per quadrant were planned in the study design. Considering the technical challenges of the procedure and the variability in the retrieved tissues, five cortical slices, 1.1 mm thick and 35 mm long, were obtained from each quadrant (Figure 1VI, Figure 2b) so that the fifth could be used as a spare slice in case of technical issues with one of the other four slices. Each cortical slice was individually wrapped in saline-moistened gauze and frozen at −20 °C.

### 2.2. Machining of Flat Dumbbell-Shaped Cortical Tissue Specimens

Each cortical slice was thawed separately. The slice was clamped on the x–y table of a milling machine with the longitudinal direction aligned with the *x*-axis of the milling machine (ProLight 1000, Light Machines Corporation, Manchester, NH, USA). The cortical slice was kept immersed in a saline solution during machining. The slice was contoured using a 3 mm diamond-coated cylindrical tool to obtain a flat dumbbell-shaped specimen with a narrow section 4 mm wide and 10 mm long. The first contour, i.e., the periosteal side of the narrow section, was machined 0.7 mm inside the original periosteal surface (Figure 1VII). The second contour, on the endosteal side, may not be completely inscribed into the cortical tissue. Therefore, before removing the specimen from the milling machine, the endosteal side of the narrow part was observed using a handheld lens with 10× magnification. If the endosteal side appeared discontinuous in the narrow part of the specimen, this side was re-contoured by decreasing the width of the narrow section by 0.5 mm. The two surfaces of the flat dumbbell specimen were then polished (Figure 1VIII). The polishing of each surface reduced the thickness of the specimen by 0.05 mm. Polishing was carried out under constant water irrigation using a micro grinder (EXAKT 400CS, EXAKT Advanced Technologies GmbH, Norderstedt, Germany) loaded with 1200-grit sandpaper. Polishing was repeated on the other side to obtain a double-sided polished dumbbell-shaped specimen with a nominal thickness of 1 mm (Figure 1IX, Figure 2c).

The fifth slice from each quadrant (referred to as a spare slice) was machined only in instances where an issue arose in managing or testing any of the four slices from the corresponding quadrant (refer to the Section 3 for details). The thickness and width of the narrow section were measured at three different levels using a digital calliper. The mean value was used to calculate the cross-sectional area of the narrow part of the specimen.

### 2.3. Mechanical Testing

After machining and before the mechanical test, each dumbbell specimen was further kept in saline solution for 1 h. Each end of the specimen was then attached to a miniaturised carriage mounted on a linear rail. The longitudinal axis of the specimen was aligned with the axis of the rail. The narrow part of the specimen was placed equidistantly from the two carriages, spaced 20 mm apart. The linear guide with the bone tissue specimen was aligned vertically with the actuator axis of a material testing machine (Mod. 8502, Instron, Norwood, MA, USA). The lower carriage was fixed to a load cell with an accuracy of 0.5% (5 kN load cell, Mod. 2518-103, Instron, Norwood, MA, USA). The upper carriage was connected to the actuator via a precision double universal joint.

A preload of 10 N was applied to the specimen. Two extensometers (Mod. 2620-601, Instron Corp., Canton, MA, USA) were simultaneously attached to the two 4 mm wide sides of the narrow part of the dumbbell specimen by means of four rubber bands. The nominal distance between the two arms, with the pin inserted in each extensometer, was 8.75 mm. However, once the two reference pins were removed, the effective initial distance between the two arms of each extensometer was measured and used to calculate the longitudinal strain on each side of the cortical bone specimen.

Monotonic tensile testing was performed under displacement control at a constant displacement rate of 2 mm/s, leading to a nominal strain rate of 0.1 s−1, i.e., slightly higher than that measured in vivo during strenuous activities (see [18] for a brief review). The signals from the load cell and the two extensometers were acquired at a frequency of 5 kHz during the test.

### 2.4. Data Processing and Analysis

An eight-point moving average filter was used to reduce signal noise collected from the three transducers, i.e., the load cell and the two extensometers.

The stress value, i.e., the measured load value divided by the cross-sectional area, was plotted against the mean value of the two longitudinal strain values calculated using the elongation measured by each extensometer. The stress–strain dataset obtained for each specimen was automatically processed to calculate the elastic modulus, the yield stress, and the yield strain.

To ensure the elastic modulus was calculated in the linear part of the stress–strain curve, (i) each data pair for which the stress value was smaller than 5 MPa was first discharged to disregard any non-linearity in the lower part of the stress–strain curve and (ii) a backward linear regression filter was used, starting from the maximum stress value, with a threshold of 0.999 on the coefficient of determination R2. The slope of the resulting regression line was taken as the elastic modulus of the tested specimen. The yield point was then defined using the 0.2% offset method applied to the previously calculated regression line (see Figure S1 in the Supplementary Materials Section).

The Kruskal–Wallis test was first used (i) to assess differences in yield strain values among individuals (*n* = 16 per individual) and, separately, around the four anatomical quadrants (*n* = 20 per quadrant) and then (ii) to assess differences in the elastic modulus and yield stress/strain values around the four anatomical quadrants. Where necessary, a non-parametric post hoc test (Bonferroni test adjusted for multiple comparisons) was used to detect statistical differences between group pairs.

The existence of a correlation between the calculated parameters was also investigated. The statistical significance of the regression model was determined and, when a statistical significance was found, the R2 value was calculated.

## 3. Results

Valid results for 80 specimens were obtained, achieving the desired sample size (*n* = 4 for each quadrant of each of the five donors). In 10 cases, it was necessary to resort to the fifth spare slice machined from each quadrant for the following reasons:

- Error in setting up the micro grinder, thus achieving a too-thin specimen (*n* = 3). In those cases, the specimen was unusable for testing;
- Error in attaching the two extensometers to the narrow part of the dumbbell specimen, thus causing the knife edges to slip on the specimen surface (*n* = 3). In those cases, the stress–strain curve showed several small drops in strain values (fine horizontal saw-tooth pattern). Additionally, no data subset satisfying the R2 ≥ 0.999 linearity requirement could be identified by the script;
- Early failure of the specimen at a low (<80 MPa) stress value (*n* = 4). In all these cases, a macroscopic discontinuity in the cortical bone tissue was found on the fracture surface.

The average values of thickness and width in the narrow cross-section of the 80 specimens were 1.00 mm (range: 0.93–1.07 mm) and 3.97 mm (3.90 mm–4.07 mm), respectively, except for the 9 specimens that required re-contouring of the endosteal region, whose average width was 3.47 mm (range: 3.40–3.57 mm).

**Figure 3:**
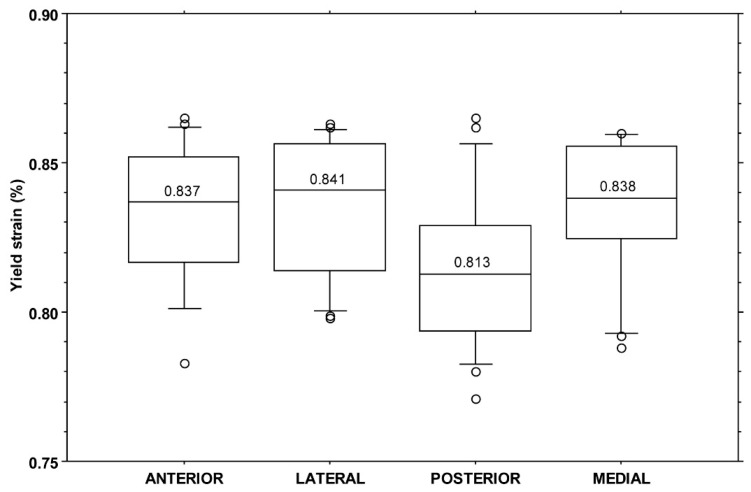
Boxplot of yield strain values (%) by quadrant. The central line in each box indicates the median value, the boxes represent the 25th and 75th percentile, and the whiskers represent the 10th and 90th percentiles.

All yield strain values fell in the range of 0.77–0.87%, with a median value of 0.83% (IQR: 0.04%). No significant difference was found among yield strain values when split by individual (KW: *p* = 0.20) or by quadrant (KW: *p* = 0.06, Figure 3).

**Figure 4:**
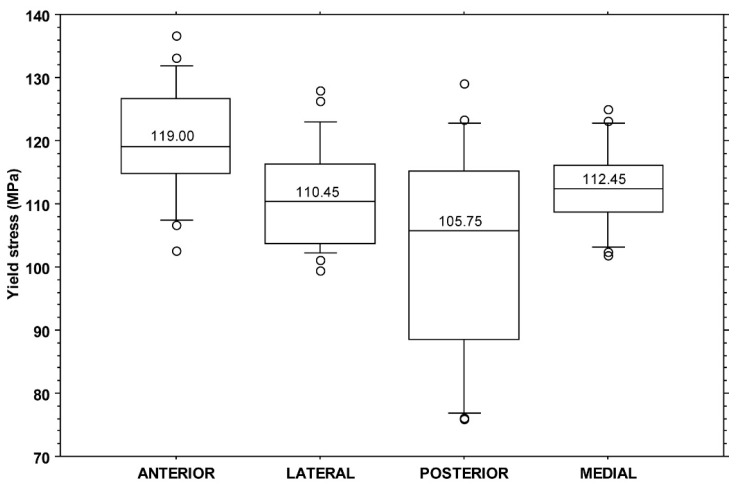
Boxplot of yield stress values (MPa) by quadrant. The central line in each box indicates the median value, the boxes represent the 25th and 75th percentile, and the whiskers represent the 10th and 90th percentiles.

All yield stress values fell in the range of 75.9–136.6 MPa with a median value of 112.5 MPa (IQR: 14.3 MPa). A significant difference was found among yield stress values when split by individuals (KW: *p* < 0.001) or by quadrants (KW: *p* = 0.001). The post hoc analysis showed a difference between the anterior and posterior (*p* < 0.001) and between the anterior and lateral (*p* = 0.026) quadrants (Figure 4).

**Figure 5:**
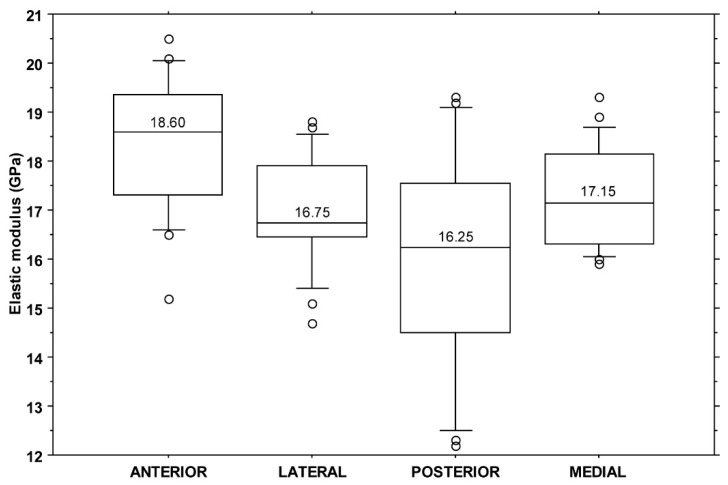
Boxplot of elastic modulus values (GPa) by quadrant. The central line in each box indicates the median value, the boxes represent the 25th and 75th percentile, and the whiskers represent the 10th and 90th percentiles.

All elastic modulus values fell in the range of 12.2–20.5 GPa with a median value of 17.2 GPa (IQR: 2.2 GPa). A significant difference was found among elastic modulus values when split by individuals (KW: *p* < 0.001) or quadrant (KW: *p* = 0.003). The post hoc analysis showed a difference between the anterior and posterior (*p* = 0.002) and between the anterior and lateral (*p* = 0.034) quadrants (Figure 5).

**Figure 6:**
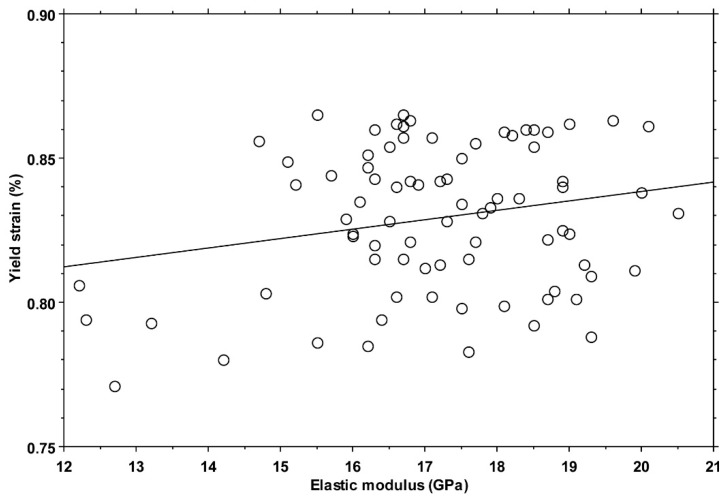
Scatterplot of yield strain versus elastic modulus for all 80 specimens. The line represents the correlation mentioned in the text.

An extremely weak positive correlation (ANOVA: *p* = 0.045, slope = 0.003%/GPa, R2 = 0.05) was found between the values of yield strain and elastic modulus (Figure 6).

**Figure 7:**
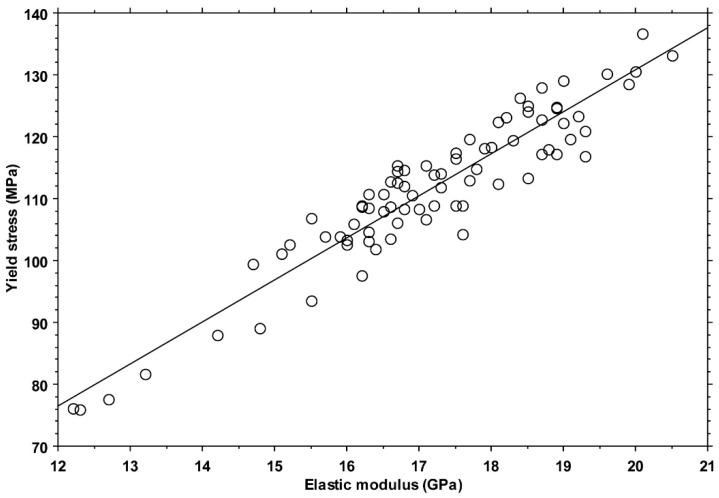
Scatterplot of yield stress versus elastic modulus for all 80 specimens. The line represents the correlation mentioned in the text.

A strong positive correlation (ANOVA: *p* < 0.001, slope = 6.8 MPa/GPa, R2 = 0.89) was found between the yield stress and elastic modulus values (Figure 7).

All the stress–strain curves, as well as the average stress–strain curve calculated for each quadrant, are shown in Figure S2 (see the Supplementary Materials Section).

## 4. Discussion

This study aimed to test the hypothesis that the yield strain values of human cortical bone measured during tensile tests are invariant among individuals and anatomical quadrants. The following important limitations must be acknowledged before discussing the present findings: (i) the number of individuals was five. This limitation is due to the difficulties in collecting bone segments from donors with no history of musculoskeletal disorders. (ii) All femurs were retrieved from adult (middle-aged) males. This choice was made to make the small group of individuals as homogeneous as possible. (iii) The cortical bone tissue specimens were collected from a single anatomical region. The reason for this choice was the identification of a femoral region where the cortical bone tissue of each quadrant is subjected to different habitual loading conditions. (iv) No density or microstructural measurements (such as those performed on microCT images) were undertaken on the specimens. (v) The number of dumbbell specimens obtained from each quadrant was small, despite the small cross-sectional dimensions—the thickness of 1 mm is a compromise between being large enough to encompass the structure of the cortical bone tissue (five times the average osteon diameter) and being small enough to allow for five slices to be obtained from each quadrant. The statistical analysis was therefore carried out separately among individuals and quadrants to ensure an adequate size of subgroups.

Considering these limitations, the collected data corroborate the hypothesis that the yield strain of cortical bone tissue, retrieved from the femoral diaphysis of adult individuals, is invariant among quadrants, i.e., to the loading condition habitually experienced. This result further corroborates the choice of a fixed yield strain value in computational models, where the elastic modulus is instead inhomogeneous and linked to density. Indeed, even considering the weak positive correlation with the elastic modulus, an increase of 5 GPa in the elastic modulus—from 15 to 20 GPa, a typical range for cortical bone tissue [16,27]—would result, according to the linear regression equation, in an increase in the yield strain of 0.015%. This increase is lower than the IQR reported in this study (overall and by quadrant) and negligible compared to the differences found among different studies.

The invariance in the yield strain of cortical bone has been already reported [15,16], although not for tissue subjected to different habitual stress states. Our median yield strain value is about 10% and 20% higher than those reported by Bayraktar et al. [15] and by Mirzaali et al. [16], respectively. Two different reasons may explain this difference as follows: (i) in the present study, the donors were middle-aged, whereas the donors in the mentioned studies were elderly (mean age: 71.8 years in the study by Bayraktar et al.; median age: 77 years in the study by Mirzaali et al. [16]). It has been shown that bone turnover decreases with age, changing the collagen phase [28,29]. An early work by McCalden et al. [30], testing human femoral cortical bone in tension, reported a marked (r < −0.70, *p* < 0.01) reduction in tissue strength and toughness with age. That same work also reported a minor but significant effect of age on tensile yield strain (r = −0.25, *p* < 0.05). This is coherent with the results of Leng et al. [31], who reported a reduction of over 10% (going from middle-aged to elderly) for compressive yield strain. (ii) Differences in the experimental procedure may have determined a different response. It is unlikely that specimen thinness influenced the measurements, as the minimum 1 mm thickness was chosen to be five times larger than the average osteon diameter. On the other hand, the experimental procedures are similar, i.e., they all involve the use of extensometers attached directly to the specimen to measure the tissue elongation. In this study, the use of two extensometers reduced the scatter of yield strain values.

If not explained by systematic differences arising from experimental procedures, the range of mean yield strain values (from 0.68% in Mirzaali et al. [16] to 0.83% in the present study) is large enough to challenge the choice of a fixed yield strain limit in computational models and calls for further, properly sized studies to test the existence of a relationship between yield strain and age. From a wider perspective, it is noteworthy that the median value for yield strain found in this study (0.83%) is close to the mean value (0.85%) calculated using equation determined by Currey (Equation 1 of the mentioned paper) after testing cortical bone tissue specimens retrieved from 23 species [32]. The median value found in this study is further supported by a study on the evolution of material properties of cortical bone [33]. In that study, the authors concluded that “*material properties of the first long bones 475 million years ago were conserved throughout evolution*”, reporting a mean value of 0.84% for yield strain among different species that can be considered representative of stepwise animal evolution.

It could be argued that our study design is not sensitive to small differences in yield strain values. However, the narrow range of measured yield strain values (0.77–0.87%) compared with the wide range of elastic modulus and yield stress values (12.2–20.5 GPa and 75.9–136.6 MPa, respectively) supports the sensitivity of the experimental design. Moreover, the significant differences among quadrants found in both the elastic modulus and yield stress values are supported by previous works. In fact, both regional and inter-individual differences in the tissue porosity and mineral density of cortical bone tissue of human femurs have been reported [34,35,36,37,38], leading to differences in tissue mechanical properties [20,39,40].

## 5. Conclusions

The present results confirm and extend previous findings about the invariance in tensile yield strain in human cortical bone, reporting that it does not significantly vary among anatomical quadrants subjected to different habitual loading conditions. The invariance in the yield strain values, combined with the strong positive correlation found between tensile yield stress and the elastic modulus, further corroborates the hypothesis put forward in comparative studies that bones adapt by changing shape, density, and architecture but not intensive material properties. The median strain value found in this study (0.83%) is very similar to what was reported in inter-species studies, but it was 10 to 20% higher than that reported in previous human studies, possibly because of the younger age of the tested subjects. This aspect warrants further investigation, also considering the rather common adoption of a fixed yield strain value in computational models.

## Data Availability

Data supporting reported results are available in the Supplementary Materials.

## Supplementary Materials

The following supporting information can be downloaded at: https://www.mdpi.com/article/10.3390/bioengineering11040395/s1, Figure S1: The 0.2% offset method; Figure S2: Aggregated stress-strain curves; Table S1: Raw data.
