# Supplementary material for: Tensile Yield Strain of Human Cortical Bone from the Femoral Diaphysis Is Constant among Healthy Adults and across the Anatomical Quadrants
Source: Bioengineering (Basel). 2024 Apr 19;11(4):395. doi: 10.3390/bioengineering11040395 (PMC11048186; doi:10.3390/bioengineering11040395)
Supplement: Supplementary file 1 [file bioengineering-11-00395-s001.zip › Baleani - Suplementary materials.pdf]

Supplementary materials

Calculation of mechanical parameters

The load cell and the two extensometers measured the load applied to the cortical bone specimen and the elongation of the narrow section. The stress (σ) was calculated using the Equation S1:

$$\sigma = F/A \qquad \text{(S1)}$$

where F is the force acting on the specimen and A is the cross-sectional area of the narrow part of the specimen. The strain (ε) was calculated using the Equation S2:

$$\varepsilon = \Delta L/L_0 \qquad \text{(S2)}$$

where ΔL is the average of the elongation measured simultaneously by the two extensometers when the force F is acting on the cortical bone specimen and L_0 initial gauge length measured with a preload of 10N.

The stress-strain curve is shown in Figure S1 (black line). The regression line fitting the linear part of the stress-strain curve is shown in the figure (red line). A second line is drawn parallel to the regression line, offset by 0.2%. The intersection of this second line (blue line) with the stress-strain curve identifies the yield point. The coordinates of the yield point are the yield strain and the yield stress.

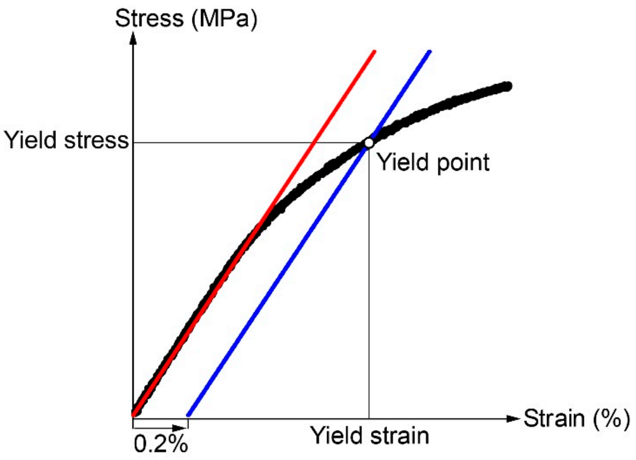

Figure S1. The 0.2% offset method. The stress-strain curve (black curve) from which the elastic modulus, yield stress and yield strain mechanical parameters are derived.

Stress-strain curves

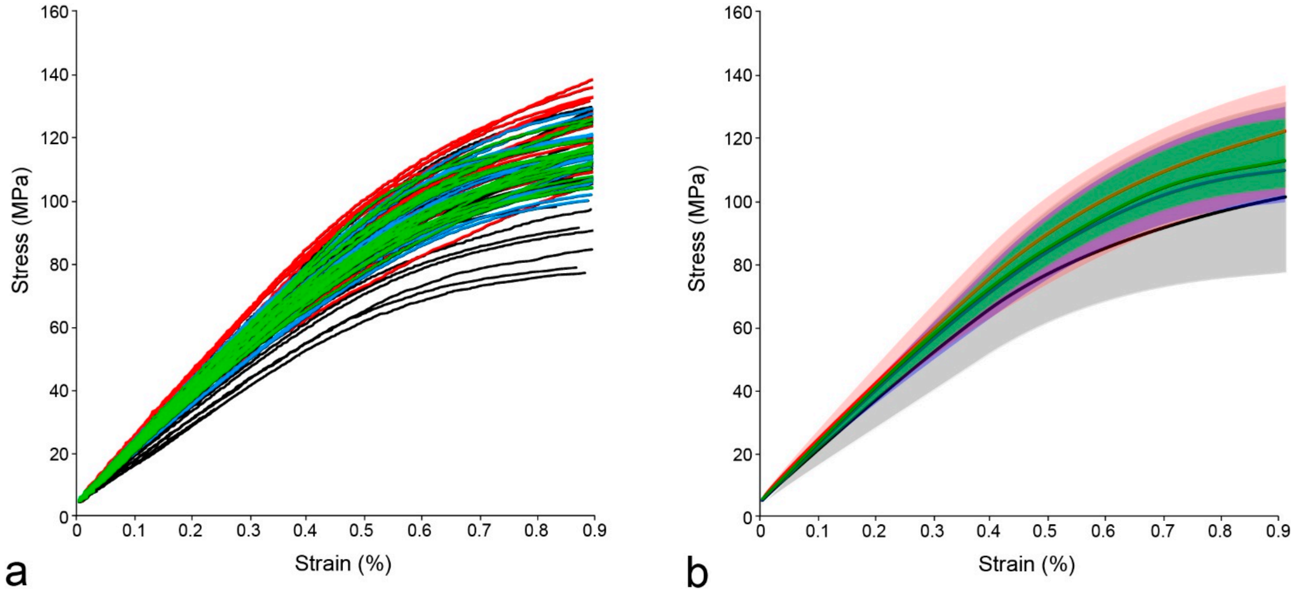

Figure S2. Aggregated stress-strain curves. (a) All the stress-strain curves calculated from the experimental data collected testing for all 80 specimens. (b) The average stress-strain curve, with the minimum-maximum band depicted around each curve, calculated for each quadrant. (Red colour = anterior quadrant; Cyan colour = lateral quadrant; Black colour = posterior quadrant; Green colour = medial quadrant). Note: the x-axis was limited to 0.9% strain to focus on the elastic regime.
