# Supplementary figures and images for: Tensile Yield Strain of Human Cortical Bone from the Femoral Diaphysis Is Constant among Healthy Adults and across the Anatomical Quadrants
Source: Bioengineering (Basel). 2024 Apr 19;11(4):395. doi: 10.3390/bioengineering11040395 (PMC11048186; doi:10.3390/bioengineering11040395)

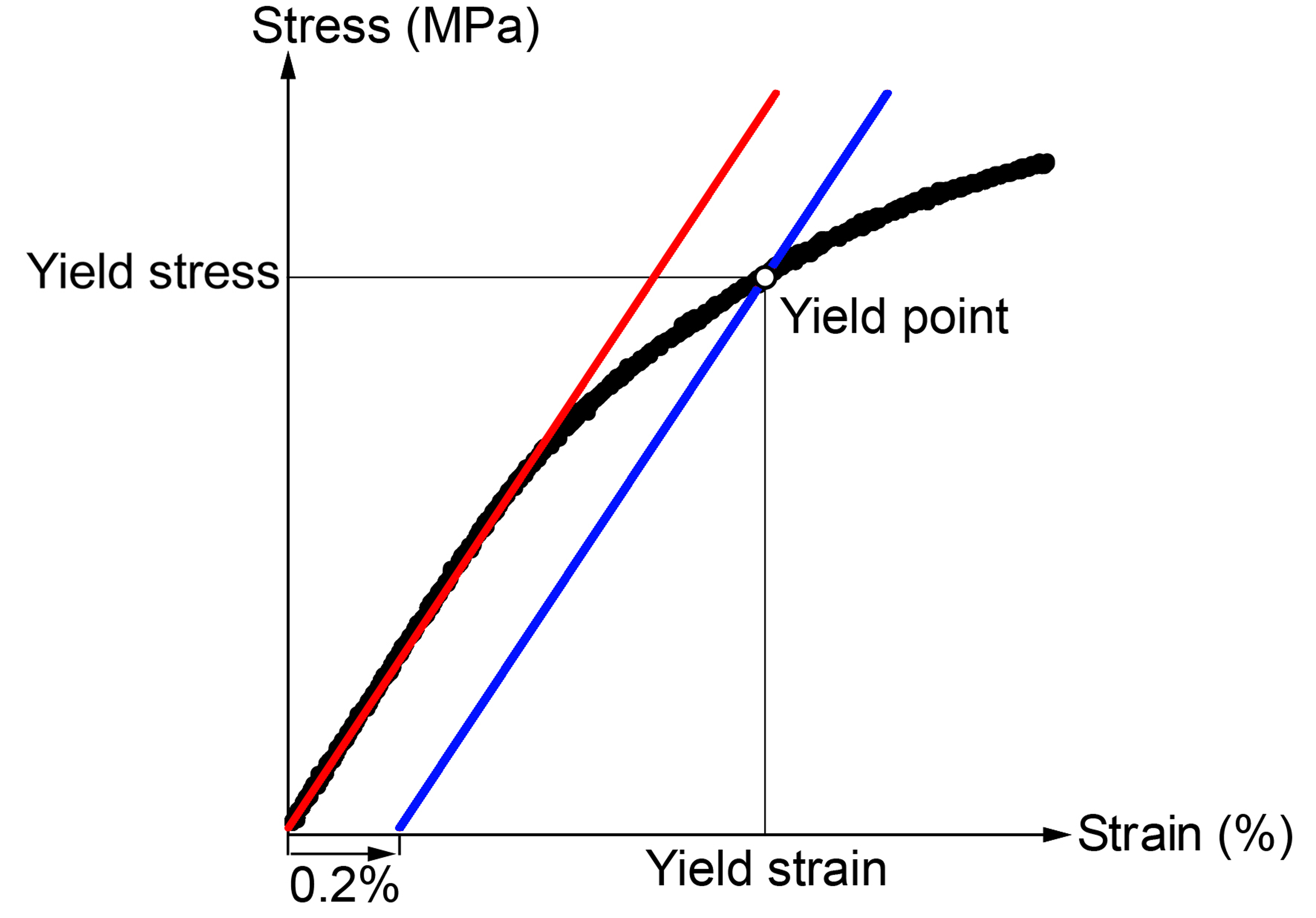

Supplement: Supplementary file 1 [file bioengineering-11-00395-s001.zip › FigureS1.jpg]

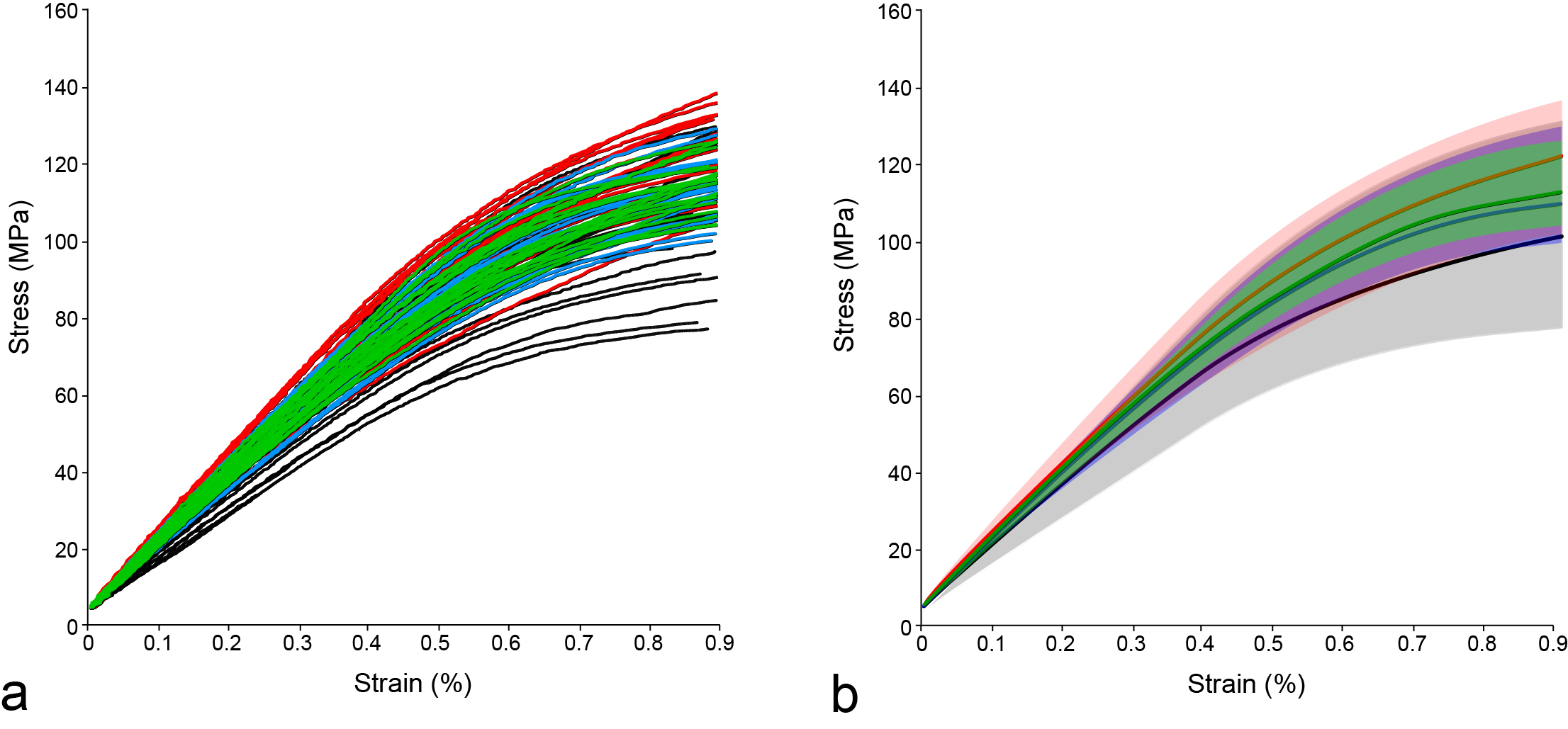

Supplement: Supplementary file 1 [file bioengineering-11-00395-s001.zip › FigureS2.jpg]
